# Saxitoxin-Equivalent Toxicity of *Gymnodinium catenatum* and Toxicity Equivalence Factors of GC Toxins Determined by the Neuro-2a Assay

**DOI:** 10.3390/toxics14090784

**Published:** 2026-09-04

**Authors:** Jeong-In Park, Jun Kim, Youngjin Kim, Young-Seok Han, Jung-Rae Rho, Yeonjung Lee, Dong Han Choi, Youn-Jung Kim

**Affiliations:** 1 Department of Marine Science, Incheon National University, Incheon 22012, Republic of Korea; qkrwjdls0812@naver.com (J.-I.P.); gottkf0167@naver.com (J.K.); yjkim@safety-as.com (Y.K.); 2 Research Institute of Basic Science, Incheon National University, Incheon 22012, Republic of Korea; 3 Department of Life Science, Hanyang University, Seoul 04763, Republic of Korea; 4 Safety Core Lab Inc., Seoul 04309, Republic of Korea; 5 NEB Co., Seoul 08054, Republic of Korea; neb.yshan@gmail.com; 6 Department of Oceanography, Kunsan National University, Gunsan 54150, Republic of Korea; jrrho@kunsan.ac.kr; 7 Ocean Climate Response & Ecosystem Research Department, Korea Institute of Ocean Science and Technology, Busan 49111, Republic of Korea; leey@kiost.ac.kr; 8 Department of Convergence Study on the Ocean Science and Technology, Ocean Science and Technology School, Korea Maritime and Ocean University, Busan 49112, Republic of Korea

**Keywords:** *Gymnodinium catenatum*, paralytic shellfish toxins, Neuro-2a assay, GC toxins, toxicity equivalence factors, voltage-gated sodium channel

## Abstract

*Gymnodinium catenatum* is a paralytic shellfish toxin (PST)-producing dinoflagellate that produces classical PSTs and hydroxybenzoate analogues known as GC toxins. Toxicological evaluation of GC toxins remains limited because certified reference materials (CRMs) and toxicity data are unavailable. This study applied the Neuro-2a (N2a) cell-based assay to evaluate PST functional toxicity in a Korean *G. catenatum* strain. The algal extract was analyzed by dose–response curve fitting to estimate total saxitoxin (STX)-equivalent toxicity, while GC1 & 2, GC3, and GC4 & 5, isolated from large-scale culture and structurally confirmed by NMR and LC-MRM-MS/MS, were evaluated to determine toxicity equivalence factors (TEFs). A clear sigmoidal curve yielded a functional toxicity of 3.7275 pg STX eq cell^−1^, and TEFs followed the order GC3 > GC4 & 5 > GC1 & 2, with potency gaps considerably larger than previously reported receptor-binding differences. These findings support the N2a assay as a complementary approach for PST analogues lacking CRMs and suggest that receptor-binding affinity alone does not fully predict cellular functional potency. Although the proposed TEFs, obtained from single assays per preparation, are preliminary and assay-specific, this study provides the first functional toxicity estimates for GC toxins, contributing to the toxicological characterization of novel PST analogues.

## 1. Introduction

Climate change-driven alterations in the marine environment are increasing the frequency and intensity of harmful algal blooms (HABs) globally. Consequently, the occurrence of paralytic shellfish toxins (PSTs) produced by toxic dinoflagellates is also becoming more frequent. Bivalves that ingest large quantities of these toxin-producing algae accumulate the toxins in their tissues. Consumption of these contaminated bivalves by humans causes paralytic shellfish poisoning (PSP). PSP occurs because PSTs selectively block voltage-gated sodium channels (VGSCs), suppressing the action potential in nerve and muscle cells [1]. Major clinical symptoms reported include numbness around the mouth, facial paralysis, and respiratory distress, and in severe cases, it can be fatal [2].

Among PST-producing dinoflagellates, *Gymnodinium catenatum* is primarily reported to produce N-sulfocarbamoyl, decarbamoyl, and carbamoyl derivatives. In addition, some regional strains have been reported to produce hydroxybenzoate PST analogues, commonly referred to as GC toxins [3,4]. Compared with classical carbamoyl PSTs, these analogues have distinct structural features that may influence their interaction with VGSCs and ultimately affect their toxic potency [5]. However, the lack of certified reference materials (CRMs) and limited toxicological information for GC toxins continues to hinder both their quantitative analysis and toxicological evaluation.

*G. catenatum* was first reported along the southern coast of Korea in 1991 [6] and has since been recognized as a toxic harmful algal species occurring sporadically in coastal waters [7]. Although no cases of PSP caused by *G. catenatum* have been recorded in Korea, related incidents have been documented in adjacent regions, including Japan and China [8,9]. With the increasing frequency of HABs under changing environmental conditions, there is a growing need to better understand the toxicity of *G. catenatum* strains occurring in Korean waters and to evaluate their potential risk to seafood safety.

Current methods for PST analysis in seafood include the AOAC mouse bioassay (MBA), high-performance liquid chromatography with fluorescence detection (HPLC-FLD), and liquid chromatography-tandem mass spectrometry (LC-MS/MS) [10,11,12,13]. Although these methods are widely used, each has limitations. MBA offers simplicity and rapidity but raises ethical concerns and has a relatively high limit of detection (LOD) of approximately 40 μg saxitoxin (STX) equivalents (eq) per 100 g of tissue [14,15]. HPLC-FLD and LC-MS/MS are highly precise analytical tools, but their application can be limited when suitable standards are not available. This is particularly problematic for poorly characterized or emerging analogues that lack CRMs.

To complement conventional analytical approaches, in vitro bioassays targeting the toxin mode of action have been actively developed [16,17]. Among these, the Neuro-2a (N2a) cell-based assay has been extensively applied to evaluate marine toxins that interact with VGSCs. Exposure of N2a cells to ouabain and veratridine induces sodium influx-dependent cytotoxicity, whereas toxins that inhibit sodium channel activity, such as PSTs, counteract this effect, resulting in a concentration-dependent increase in cell viability [18,19]. Because the assay reflects VGSC-mediated biological activity, it provides a functional measure of toxicity rather than simple chemical concentration. The N2a assay has also been reported to be highly sensitive and has been applied to a range of marine toxins that act on sodium channels [18,20,21,22].

Therefore, the present study applied the N2a assay to evaluate the functional toxicity of PSTs produced by *G. catenatum*. First, the assay conditions for the *G. catenatum* toxin extract were optimized to estimate its total STX-equivalent functional toxicity. Second, purified GC toxins obtained from the same strain were evaluated using the N2a assay to determine their EC_50_ values and to estimate toxicity equivalence factors (TEFs) relative to STX. Through this approach, we aimed to assess the applicability of the N2a assay as a complementary platform for the functional evaluation of GC toxins for which toxicological information and certified standards remain limited.

## 2. Materials and Methods

### 2.1. Reagents and Materials

#### 2.1.1. Neuro-2A Cell Culture

The N2a cell line was purchased from the American Type Culture Collection (ATCC, Manassas, VA, USA). Cells were cultured in RPMI 1640 medium (Gibco, Grand Island, NY, USA), containing 10% fetal bovine serum (FBS; Gibco, Grand Island, NY, USA), 1% penicillin-streptomycin (Gibco, Grand Island, NY, USA), 2 mM GlutaMAX (Gibco, Grand Island, NY, USA), 1% sodium pyruvate (Gibco, Grand Island, NY, USA), and 2.5 μg mL^−1^ amphotericin B (Gibco, Grand Island, NY, USA). The cultures were kept at 37 °C with 5% CO_2_. RPMI 1640 with 10% FBS was used for routine maintenance and subculturing, while RPMI 1640 with 2% or 5% FBS was used for experimental media, depending on the experimental step. Dulbecco’s phosphate-buffered saline (DPBS; Gibco, Grand Island, NY, USA) was used for cell washes.

#### 2.1.2. Toxin Standards and Reagents

The certified PST used in this study was saxitoxin dihydrochloride (STX; Sigma-Aldrich Production GmbH, Switzerland). Ouabain octahydrate (O; Sigma-Aldrich, St. Louis, MO, USA) was dissolved in sterile distilled water to prepare a 20 mM stock solution, while veratridine (V; Sigma-Aldrich, St. Louis, MO, USA) was prepared at a concentration of 5 mM using sterile distilled water adjusted to pH 2. For evaluation of cell viability, a 5 mg mL^−1^ solution of thiazolyl blue tetrazolium bromide (MTT; Sigma-Aldrich, St. Louis, MO, USA) was prepared by dissolving the powder in PBS and then filtering it through a 0.22 μm syringe filter.

#### 2.1.3. Algae Collection and Culture

The mass-cultured *G. catenatum* strain KM032 was obtained from the Korea Institute of Ocean Science & Technology (KIOST, Busan, Republic of Korea). This strain was originally isolated from Tongyeong, Republic of Korea. Cultures were grown in filtered natural seawater adjusted to 30 psu and supplemented with f/2-selenium nutrient solution (Fritz Aquatics, Mesquite, TX, USA) in 20 L culture tanks at 20 °C under a 12:12 h light:dark cycle with a light intensity of 200–250 µmol m^−2^ s^−1^ [23]. Subculturing was performed every 7 to 10 days. For algal extraction, approximately 36,855,000 cells, representing the maximum harvestable amount, were collected in a 50 mL tube and stored in a refrigerated chamber until extraction.

### 2.2. Preparation of G. catenatum Toxin Extract

Toxin extraction from *G. catenatum* was performed with modifications based on the methods of Wiese et al. (2014) [24]. The harvested cell suspension was centrifuged at 3000 rpm for 15 min at 4 °C, and the resulting cell pellet was reconstituted in 10 mL of 0.1 N HCl. This mixture was homogenized using an ultrasonicator for 10 min, followed by heating at 90 °C for 5 min. After heating, the mixture was vortexed. The suspension was then centrifuged at 3000 rpm for 15 min at 4. The supernatant was then filtered using a 0.45 μm syringe filter to obtain the final test solution. The filtered extract had a concentration of 3,685,500 cell eq mL^−1^. A 21-fold dilution was performed, resulting in a final concentration of 175,500 cells eq mL^−1^ used as the highest concentration in the N2a assay.

### 2.3. Isolation of GC Toxins from Large-Scale Culture of G. catenatum

The experimental methodology employed for the detection of GC toxins using HPLC-PCOX-FLD (e2695: Waters, Milford, MA, USA) has previously been documented by Park et al. (2024) [23]. Four GC toxins were isolated from a 500 L culture of *G. catenatum* prepared according to the protocol described above. Cell pellets harvested via continuous centrifugation were extracted with 1 L of 0.1 N HCl and subsequently partitioned twice against with dichloromethane (CH_2_Cl_2_) to remove lipophilic contaminants. The resulting aqueous layer was loaded onto a glass column (3.0 × 90 cm) packed with Bio-Gel P-2 media (Fine 45–90 µm wet; Bio-Rad, Hercules, CA, USA) at a flow rate of 1.0 mL/min and 10 mL fractions were collected using an automated fraction collector (DC-1500, EYELA, Tokyo, Japan). Fractions containing GC toxins (eluted after approximately 3 h; total volume 240 mL) were pooled and concentrated. The enriched extract was further separated by semi-preparative HPLC (Agilent 1200 series, Agilent Technologies, Santa Clara, CA, USA) equipped with a TSK-Gel Amide-80 HILIC column (7.8 mm i.d. × 250 mm, 5 µm, Tosoh Bioscience, Shunan, Japan). The mobile phases consisted of (A) H_2_O and (B) acetonitrile, both containing 2 mM ammonium formate and 0.1% formic acid. Separation was performed at a flow rate of 2.0 mL/min using a linear gradient: 75% B to 45% over 17 min, followed by isocratic elution at 45% B for 25 min. Two subfractions were obtained: Subfraction I (5 mg, tR = 18 min, containing GC1 & 2, and GC 4 & 5) and Subfraction II (2.5 mg, tR = 23 min, containing GC3). To achieve final purification of individual toxins, Sub-fractions I and II were separately subjected to reversed-phased HPLC on a Polar C18 column (Phenomenex, Torrance, CA, USA) under isocratic conditions with 95% H_2_O and 5% MeOH containing 2 mM ammonium formate at a flow rate of 2.0 mL/min, yielding purified GC1 & 2 (0.4 mg), GC3 (0.5 mg) and GC4 & 5 (0.5 mg). The purity of each isolated GC toxin was determined by HPLC-FLD, calculated as the percentage of the peak area corresponding to the target GC toxin relative to the total peak area of the chromatogram, and was estimated as >90% for all. Representative HPLC-FLD chromatograms for each purified toxin are provided in Figure S1. For the N2a assay, the toxins were prepared to achieve the highest practical concentration for testing.

### 2.4. MS Analysis for Identification and Tracking of GC Toxins

Target GC toxins during the isolation procedure were tracked and confirmed by LC-MS/MS using a SCIEX 3200 QTRAP mass spectrometer (SCIEX, Framingham, MA, USA) equipped with an electrospray ionization (ESI) source operating in positive ionization mode. Chromatographic separation was achieved on a TSK-Gel Amide-80 column (2.1 mm i.d. × 150 mm, 5 µm; Tosoh Bioscience, Shunan, Japan) using mobile phase (A) H_2_O and (B) acetonitrile, each containing 5 mM ammonium formate and 0.1% formic acid. The gradient profile was programmed as follows at a flow rate of 0.3 mL/min: 95% B to 50% B over 9 min, held at 50% B for 10 min, and re-equilibrated to initial conditions. Multiple reaction monitoring (MRM) transitions were optimized for specific precursor-to-product ion pairs: *m*/*z* 473.2 → 392.2 for GC1, *m*/*z* 473.2 → 375.2 for GC2, *m*/*z* 377.1 → 204.1 for GC3, *m*/*z* 489.2 → 391.1 for GC4, and *m*/*z* 489.2 → 409.2 for GC5.

### 2.5. Neuro-2A (N2a) Assay

#### 2.5.1. Methodology of the N2a Assay

**Figure 1:**
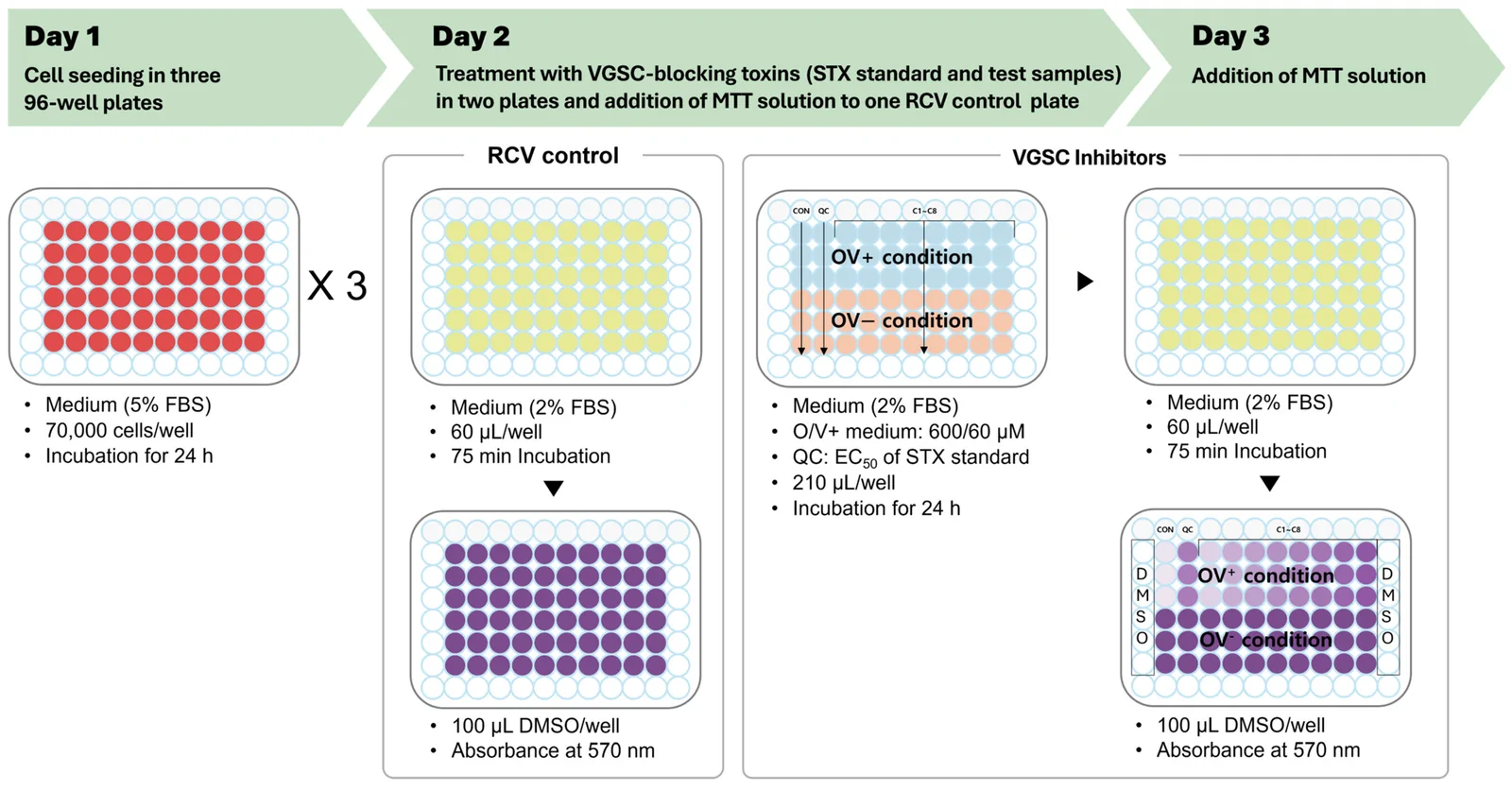
Schematic overview of the Neuro-2a assay workflow used for evaluating *G. catenatum* extracts and purified GC toxins.

We used the N2a assay, primarily based on the method proposed by Viallon et al. (2020) [25], with several modifications. N2a cells were detached by trypsinization and resuspended in RPMI 1640 medium with 5% FBS. The cells were seeded at 70,000 cells per well in 96-well plates and incubated for 24 h at 37 °C. A schematic overview of the assay workflow is shown in Figure 1. To reduce evaporation-related edge effects during incubation, sterile tertiary distilled water was added to the outermost cell-free wells.

For dose–response analysis and determination of EC_50_ values, all test samples, including the STX standard, *G. catenatum* toxin extract, and purified GC toxins, were prepared at the highest practical concentrations and serially diluted in eight 1:2 steps. Each diluted sample was mixed with RPMI 1640 medium with 2% FBS to produce the final test solution, corresponding to a 21-fold dilution from the original stock solutions. Two exposure conditions were prepared: untreated medium (OV−) and treated medium (OV+). The OV+ medium contained ouabain and veratridine at final concentrations of 600 and 60 μM, respectively. For each assay plate, the STX standard was tested in parallel with the corresponding test sample, and both OV− and OV+ conditions were arranged on the same plate in three rows each. After preparation of the exposure mixtures, 210 μL was dispensed into each well, and the plates were incubated for an additional 24 h. Because sample availability was limited, each dose–response experiment was conducted on a single 96-well plate, with triplicate technical wells for each concentration.

Quality control (QC) criteria were established following Viallon et al. (2020) [25] to improve consistency across plates. These criteria included reference cell viability (RCV), viability controls under OV− and OV+ conditions (COV− and COV+), and quality control samples under OV− and OV+ conditions (QCOV− and QCOV+). The EC_50_ value of 2861 fg μL^−1^ for STX, as proposed by Kim et al. (2024) [26], was used as the QC reference value. The definition, acceptance criterion, and biological interpretation of each QC parameter are summarized in Table 1. Only plates that satisfied all QC requirements were retained for data analysis, and all data reported in this study were obtained from plates that passed these QC criteria.

Following incubation, the treatment medium was removed. Then, 60 μL of working MTT solution, prepared by mixing a 5 mg mL^−1^ MTT stock with 2% FBS medium at a 1:6 ratio, was added to each well, and the plates were incubated for 75 min. After removal of the reaction medium, 100 μL of DMSO was added to solubilize the resulting formazan crystals. Optical density was measured at 570 nm using a Varioskan LUX Multimode Microplate Reader (Thermo Fisher Scientific, Waltham, MA, USA).

#### 2.5.2. Data Analysis and EC_50_ Determination

Raw absorbance values measured at 570 nm were converted to net absorbance values by subtracting the mean absorbance of DMSO-treated wells, which was approximately 0.04, from each well. In accordance with the QC criteria adopted from Viallon et al. (2020) [25], all assay results were analyzed and expressed as absorbance values rather than normalized cell viability.

The EC_50_ value was analyzed using Prism 9 Software (GraphPad, San Diego, CA, USA) based on the four-parameter logistic regression model shown in Equation (1). In this model, Y denotes the net absorbance value and X represents the tested concentrations. Dose–response curves were fitted by four-parameter logistic nonlinear regression (4PL) after log10 transformation of toxin concentrations.

Y = Bottom + (Top − Bottom)/(1 + 10^((Log EC_50_ – Log *X*) × Hillslope)) (1)

Because OV− conditions were used only to assess non-specific cytotoxicity of the test samples, OV− data were not used for EC_50_ estimation. Nonlinear regression analysis was performed only on the OV+ data, in which VGSC-blocking activity produced concentration-dependent changes in cell viability.

Best-fit values for EC_50_, Top, Bottom, and Hill slope were obtained from the fitted curves. For each dose–response curve, the best-fit EC_50_ value and its 95% confidence interval (95% CI) were derived from the nonlinear regression model. The EC_50_ corresponds to the concentration at the midpoint between the Bottom and Top values on the fitted curve.

For toxin quantification of the *G. catenatum* toxin extract, only dose–response curves showing an adequate sigmoidal pattern across the tested concentration range were considered acceptable, following the criteria described by Viallon et al. (2020) [25]. In practice, this required that at least three concentration points fell below 20% or above 80% of the response range in the high or low concentration regions, respectively. When the initial curve did not meet these criteria, further dilutions (1/10 and 1/20) were prepared, and the N2a assay was repeated to ensure the adequacy of the results.

For consistency of data presentation, EC_50_ values were rounded to one decimal place, whereas STX equivalent toxicity values and TEFs were rounded to four decimal places. Only plates that satisfied all predefined QC criteria were considered valid and included in the final analysis. For the extract suitability test, differences between control and extract-treated groups under OV− conditions were analyzed by one-way ANOVA with Dunnett’s multiple comparison test; *p*-value < 0.05 was considered statistically significant using Prism 9.

### 2.6. Determination of Toxicity Equivalence Factors (TEFs)

The TEF is defined as the relative toxic potency of a compound that shares the same mode of action as a reference substance within the same chemical group [27,28]. TEFs are widely applied in risk assessments and comparative toxicological studies to express the toxicity of individual toxin analogues on a standardised scale [29,30,31]. For PSTs, which primarily exert their toxic effects by inhibiting VGSCs, TEFs have been extensively used in conjunction with cell-based bioassays, such as the N2a assay, to compare relative toxic potency.

In this study, we proposed TEFs for structurally distinct hydroxybenzoate PST analogues (GC1 & 2, GC3, and GC4 & 5) produced by *G. catenatum*, as determined by the N2a assay. EC_50_ values were determined for each purified GC toxin and the reference toxin, STX. TEFs were calculated relative to STX, which was assigned a TEF value of 1.0 in accordance with current recommendations [28]. The TEFs were calculated using Equation (2).

TEF_GC toxin_ = EC_50 STX_/EC_50 GC toxin_ (2)

For each GC toxin, the TEF was calculated using the EC_50_ value of STX obtained in the same assay batch. Thus, batch-matched STX reference curves were used for all TEF calculations. This approach assumes that all PST analogues induce cytotoxicity in the N2a assay through a shared VGSC-blocking mechanism and that the measured EC_50_ values accurately reflect their potency for VGSC-mediated neurotoxicity.

## 3. Results

### 3.1. STX-Equivalent Functional Toxicity of G. catenatum Extract in the N2a Assay

**Figure 2:**
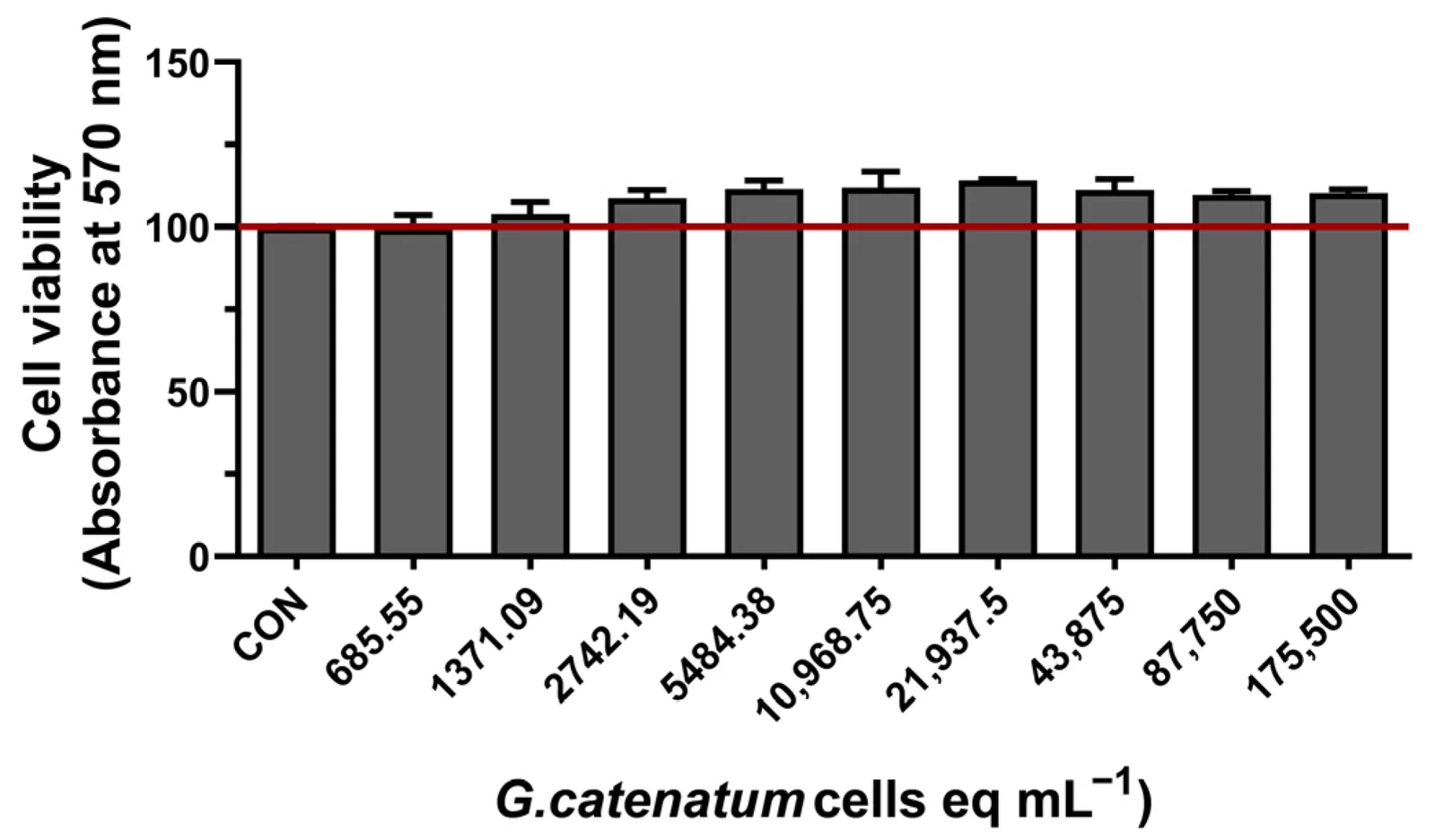
Effect of *G. catenatum* extracts on N2a cell viability measured by MTT assay under OV-conditions. Data are presented as mean ± SD of triplicate technical wells from a single experiment. The red line indicates 100% cell viability of the control group. Statistical analysis was performed using one-way ANOVA with Dunnett’s multiple comparison test against the control group. No significant difference in cell viability was observed at any tested concentration compared with the control (*p* > 0.05).

When applying the N2a assay to algal extracts, matrix components other than VGSC-inhibiting toxins may induce non-specific cytotoxicity or otherwise interfere with assay performance, including alteration of the toxin-dependent response under OV+ conditions [32,33,34]. Because such matrix effects can compromise the reliability of toxin detection and quantitative interpretation, the suitability of the *G. catenatum* extract was first evaluated under OV− conditions. In this study, *G. catenatum* extracts obtained by extraction with 0.1 N HCl did not significantly affect N2a cell viability under OV− conditions (*p* > 0.05), indicating the absence of detectable non-specific cytotoxicity from the algal extract under the assay conditions (Figure 2).

**Figure 3:**
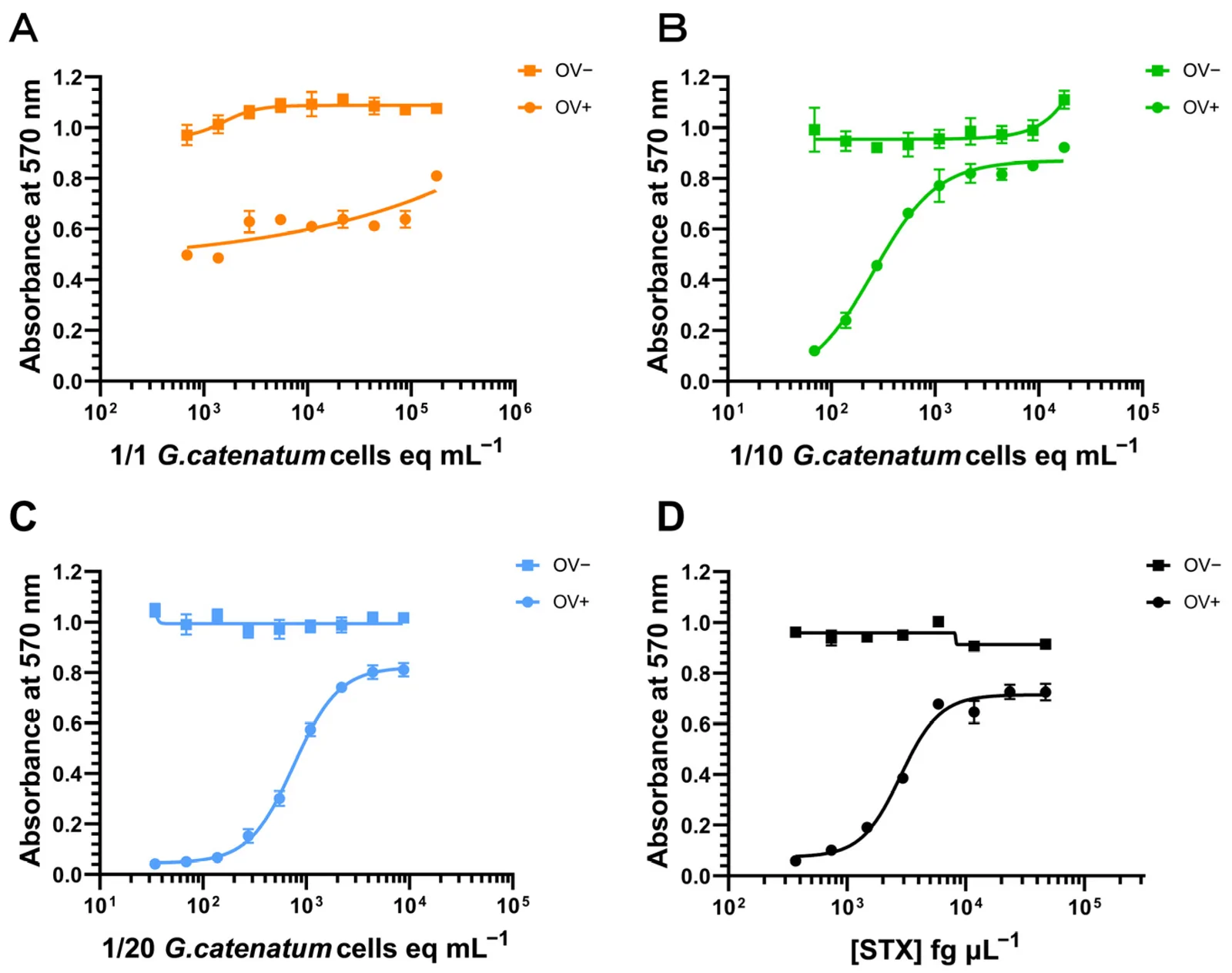
Dose–response curves obtained using the N2a assay for (**A**) the 1/1, (**B**) 1/10, and (**C**) 1/20 dilution of *G. catenatum* extract and (**D**) STX. Data are presented as mean ± SD of triplicate technical wells from a single experiment.

The extract was subsequently evaluated under OV+ conditions to identify a dilution range that produced an acceptable sigmoidal dose–response relationship for EC_50_ estimation according to the curve suitability criteria described in Section 2.5.2. The undiluted extract (1/1 extract, maximum concentration = 175,500 cells eq mL^−1^) did not produce an acceptable sigmoidal dose–response curve, and an EC_50_ value could not be calculated (Figure 3A). The extract was therefore further diluted and re-evaluated. For the 1/10 dilution (maximum concentration = 17,550 cells eq mL^−1^), the response curve remained incomplete at the lower concentration range, although an EC_50_ value of 254.2 cells eq mL^−1^ (95% CI, 164.7–317.6 cells eq mL^−1^) was obtained (Figure 3B). In contrast, the 1/20 dilution (maximum concentration = 8775 cells eq mL^−1^) produced a clear sigmoidal dose–response relationship across the full concentration range, allowing reliable estimation of the EC_50_, which was 757.2 cells eq mL^−1^ (95% CI, 709.7–808.2 cells eq mL^−1^) (Figure 3C).

To further characterize the dilution-dependent differences in curve shape, the best-fit Hill slopes and goodness-of-fit values of the OV+ dose–response curves were compared (Table 2). The undiluted extract did not yield a reliably interpretable sigmoidal fit (R^2^ = 0.639), whereas the 1/10 and 1/20 dilutions yielded Hill slopes of 1.409 (95% CI, 1.003–1.882; R^2^ = 0.985) and 1.985 (95% CI, 1.760–2.240; R^2^ = 0.996), respectively. The corresponding STX reference curve had a Hill slope of 2.615 (95% CI, 1.913–3.808; R^2^ = 0.984). The 95% CI of the Hill slope for the 1/10 dilution did not overlap with that of STX, whereas the interval obtained for the 1/20 dilution overlapped with that of the STX reference.

Because non-specific cytotoxicity of the undiluted extract had already been excluded under OV− conditions, the dilution-dependent change in curve shape observed under OV+ conditions is unlikely to reflect generalized loss of cell viability. Accordingly, the 1/20 dilution, which provided the most appropriate sigmoidal fit for quantitative interpretation, was selected for EC50-based estimation of STX-equivalent toxicity. Using STX as the reference toxin, the EC_50_ value for STX was determined to be 2822.5 fg μL^−1^ (95% CI, 2479.8–3170.4 fg μL^−1^) under the same assay conditions (Figure 3D). Based on this analysis, the functional VGSC-mediated toxicity of the strain was estimated as 3.7275 pg STX eq cell^−1^ (Table 3).

### 3.2. Structural Identification of the Isolated GC Toxins

**Figure 4:**
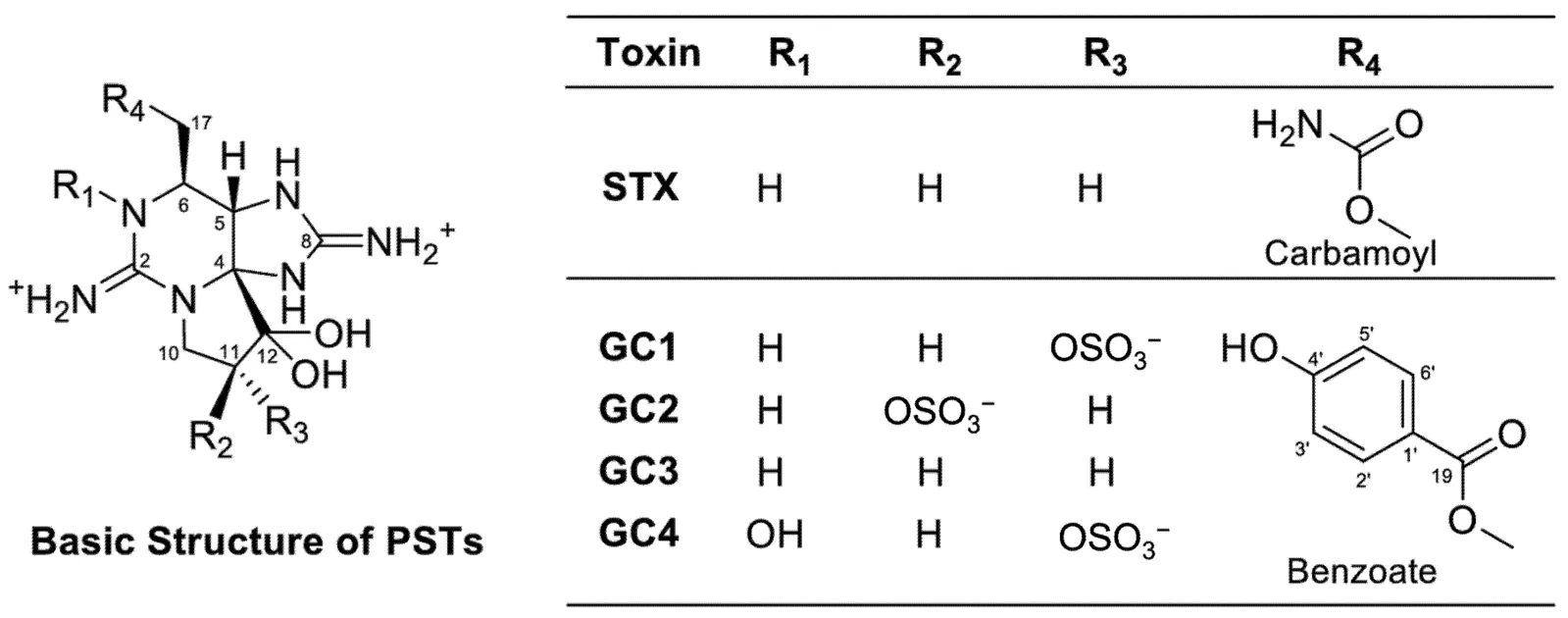
Chemical structures of STX and GC toxins isolated from *G. catenatum*.

The structures of the four isolated GC toxins were unambiguously established through spectroscopic analyses, combining 1D (^1^H) and 2D (^1^H-^1^H COSY and TOCSY) NMR spectroscopy in conjunction with LC-MRM-MS/MS. Despite minor trace impurities inherent to natural toxin isolates, well-resolved ^1^H NMR spectra were acquired in D_2_O containing 0.1% acetic acid-d4 (Figures S2–S4), and all observable proton resonances were assigned as summarized in Table 4. Notably, the H-5 signals were not observed in any of the four GC toxins under the standard acquisition temperature (25 °C). While its theoretical resonance near 4.9 ppm overlaps with the residual HOD solvent peak [35], the absence of detectable ^1^H-^1^H COSY cross-peaks and vicinal coupling to H-6 reflects severe line-broadening. This phenomenon is attributable to intermediate chemical exchange and exchange-mediated relaxation governed by rapid protonation/tautomeric equilibria of the adjacent guanidinium moieties. The chemical shifts and coupling constants of GC1 & 2 were nearly identical to previous literature values [35,36]. The proton spin systems for GC3 and GC4 & 5 were systematically assigned based on COSY and TOCSY correlations (Figures S5 and S6). In parallel, the specific MRM fragmentation patterns obtained from LC-MS/MS analysis (Figure S7) corroborated the identities and chromatographic homogeneity of all four isolated GC toxins. The chemical structures of STX and the four GC toxins isolated in this study (GC1–GC4), together with their R1–R4 substituent assignments established from NMR and LC-MRM-MS/MS data, are summarized in Figure 4.

### 3.3. Relative Toxic Potency of Purified GC Toxins Determined by the N2a Assay

**Figure 5:**
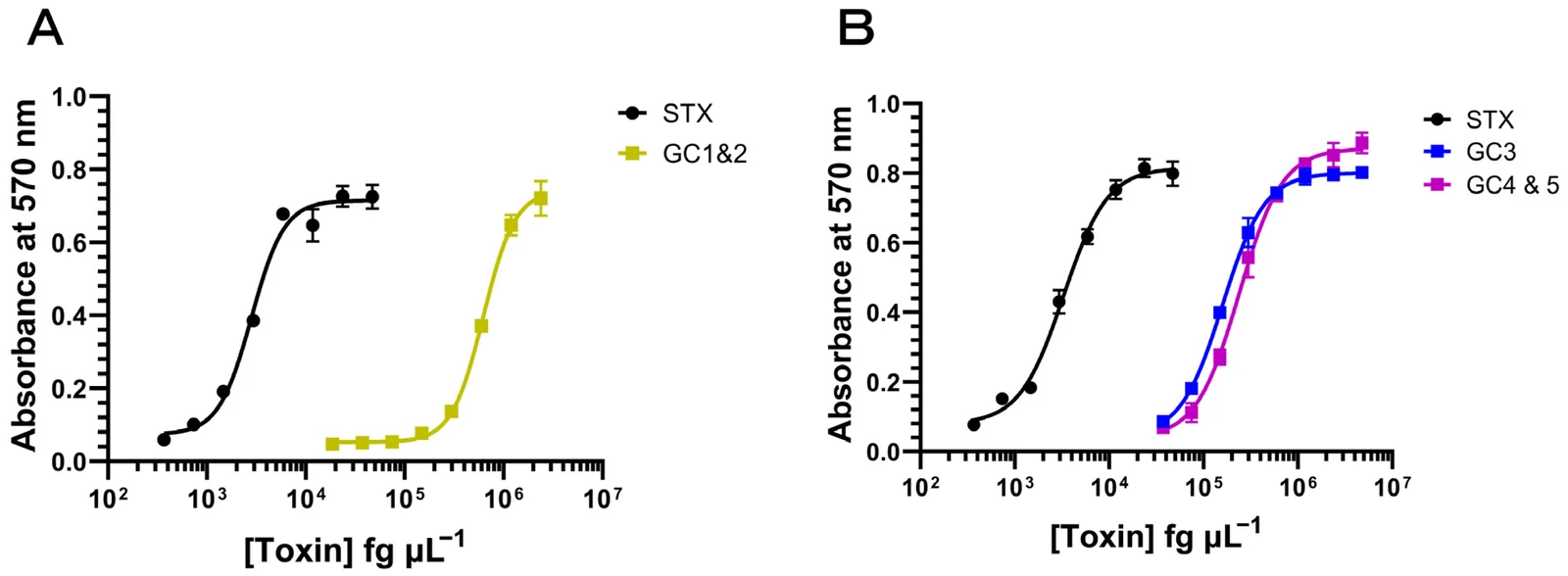
Dose–response curves obtained using the N2a assay for STX and GC toxins under OV+ conditions. (**A**) Assay 1 included STX and GC1 & 2. (**B**) Assay 2 included STX, GC3, and GC4 & 5. Data are presented as mean ± SD of triplicate technical wells from a single experiment for each assay.

To evaluate the relative functional potency of GC toxins, GC1 & 2, GC3, and GC4 & 5 were tested in the N2a assay at the highest practical concentrations achievable in the assay medium. The maximum tested concentration was 2,381,377 fg μL^−1^ for GC1 & 2 and 4,762,753 fg μL^−1^ for GC3 and GC4 & 5. All purified GC toxins produced sigmoidal dose-response curves, allowing EC_50_ values to be determined reliably (Figure 5). The EC_50_ values were 623,809 fg μL^−1^ (95% CI, 587,544–665,583) for GC1 & 2, 159,151 fg μL^−1^ (95% CI, 146,764–171,143) for GC3, and 238,384 fg μL^−1^ (95% CI, 214,913–262,881) for GC4 & 5.

To derive TEFs, the EC_50_ of each toxin was compared with the EC_50_ of STX obtained in the same assay batch. The EC_50_ value for STX was 2822.5 fg μL^−1^ (95% CI, 2479.8–3170.4 fg μL^−1^) in Assay 1 and 3278.2 fg μL-1 (95% CI, 2922.9–3667.8 fg μL^−1^) in Assay 2. Accordingly, the STX EC_50_ from Assay 1 was used for GC1 & 2, whereas that from Assay 2 was used for GC3 and GC4 & 5. Based on these batch-matched comparisons, the TEFs relative to STX were estimated to be 0.0045 for GC1 & 2, 0.0206 for GC3, and 0.0138 for GC4 & 5, with the relative functional potency following the order GC3 > GC4 & 5 > GC1 & 2 (Table 5).

## 4. Discussion

The estimated functional toxicity of 3.7275 pg STX eq cell^−1^ indicates that the Korean *G. catenatum* strain evaluated in this study produces measurable levels of VGSC-blocking toxins that can be quantified functionally using the N2a assay. In this respect, the N2a assay provides an integrated measure of biological activity rather than concentration alone. This technique is particularly useful when toxin profiles are complex or when some analogues cannot be accurately quantified due to the lack of CRMs. The present result, therefore, supports the use of the N2a assay as a complementary approach for evaluating the functional neurotoxicity of *G. catenatum* extracts.

The dilution series (1/1, 1/10, and 1/20) evaluated under OV+ conditions (Section 3.1) was designed as an assay optimization step to identify the dilution ratio that would yield a dose–response curve suitable for EC_50_ estimation. Because non-specific cytotoxicity of the undiluted extract had already been excluded under OV− conditions (Figure 2), the observed dilution-dependent improvement in curve shape is unlikely to reflect generalized cell death. It is consistent with, although does not demonstrate matrix-related interference in the O/V-sensitized, VGSC-mediated response. Such interference may arise from non-toxin matrix components co-extracted with the toxins. This interpretation is consistent with Aballay-González et al. (2016) [32], who reported matrix-related interference in the N2a assay using PST-negative bivalve extracts, including altered responses under OV+ conditions. The present results further suggest that such interference may also manifest as a dilution-dependent alteration in the shape of the extract dose–response curve. Alternatively, the concentrated extract may contain a mixture of PST analogues with differing potencies, which could alter the composite dose–response curve independently of interference from co-extracted non-toxin matrix components. Although the present data cannot fully distinguish between these possibilities, the 1/20 dilution produced a dose–response curve with Hill slope and goodness-of-fit statistics comparable to those of the STX standard, supporting the selection of the 1/20 dilution for EC_50_ estimation under the present assay conditions.

Extraction with 0.1 N HCl and heating can induce interconversion of acid-labile PST analogues, particularly hydrolysis of N-sulfocarbamoyl toxins to the corresponding carbamoyl analogues, thereby altering the native toxin profile and potentially the composite functional potency. Accordingly, the 3.7275 pg STX eq cell^−1^ value should be interpreted as the functional toxicity of the extract generated under the present extraction conditions rather than as an unbiased estimate of the native intracellular toxin profile.

Previously reported toxicity values for *G. catenatum* strains from other regions are summarized in Table S1. These values were generated under different analytical, culture, and extraction conditions and therefore cannot be compared directly with the present estimate. Environmental factors such as temperature and salinity are also known to affect PST production and toxin composition in *G. catenatum* [37,38]. Although these factors were not examined here, they may contribute to the variability among strains and studies, underscoring the need for standardized assay conditions when comparing functional toxicity data.

Turning to the purified GC toxins, this acid-hydrolysis consideration does not directly apply, as GC1 & 2, GC3, and GC4 & 5 belong to a structurally distinct hydroxybenzoate class rather than the N-sulfocarbamoyl/carbamoyl group, and their identity and purity were independently confirmed by NMR and LC-MRM-MS/MS (Section 3.2). All tested purified GC toxins exhibited substantially lower potency than STX in the N2a assay, but clear differences were observed among them, with the relative potency following the order GC3 > GC4 & 5 > GC1 & 2. These results indicate that functional potency of hydroxybenzoate PST analogues is not uniform and may depend on structural differences among the GC toxins.

GC toxins share the basic STX scaffold but carry benzoyl-substituted moieties, differing in their substitution patterns, including sulfation and hydroxylation at specific positions. The structural assignments obtained in the present study by NMR and LC-MRM-MS/MS are summarized in Figure 4 and Table 4. Such structural variation may influence toxin interaction with VGSCs and thereby contribute to the potency differences observed among GC1 & 2, GC3, and GC4 & 5 [39]. However, the present data are insufficient to identify the individual structural features responsible for these differences. Definitive structure–activity relationships will require individually purified analogues and complementary approaches capable of directly characterizing toxin–channel interactions.

The N2a-derived potency estimates can also be considered in relation to previous receptor-binding and computational studies. Llewellyn et al. (2004) [5] reported that GC3 and the GC1/GC2 epimeric mixture showed approximately 5-fold and 20-fold lower binding potency than STX, respectively, whereas the corresponding functional potency gap observed in the present N2a assay was substantially larger (48-fold and 222-fold lower than STX, respectively). This difference suggests that receptor-binding affinity alone does not fully predict the magnitude of the functional response at the cellular level. Similarly, molecular docking approaches, such as that performed by Durán-Riveroll et al. (2016) [40], estimate toxin–channel interactions and relative binding affinities from structural models but do not necessarily reproduce the integrated cellular response measured by the N2a assay. In contrast, the N2a-based TEFs reported in the present study were derived experimentally from a cell-based functional endpoint reflecting VGSC-mediated activity rather than from computational prediction. The relative potency ranking obtained here should therefore be regarded as complementary to, rather than as confirmation or contradiction of, the docking-based predictions.

The interpretation of the GC toxin results also requires consideration of the composition of the tested preparations. GC toxins comprise a structurally broader group than the GC1–3 analogues initially characterized. Vale (2008) [41] described a more complex hydrophobic PST profile in *G. catenatum*, including the N1-hydroxylated analogues GC4–6 and two additional proposed benzoyl subfamilies, GC1a–GC6a and GC1b–GC6b, resulting in up to 18 proposed GC analogues, although many of these additional structures were tentatively assigned from chromatographic and mass-spectrometric evidence rather than unequivocally established by NMR. The present study was not designed as a comprehensive profiling analysis of the complete GC toxin repertoire of the Korean KM032 strain, but instead focused on the GC1 & 2, GC3, and GC4 & 5 that could be isolated in sufficient amounts for functional testing. Consequently, the possible presence of additional GC analogues at lower abundance cannot be excluded, and the TEFs reported here should be interpreted specifically for the tested preparations rather than as representative of the entire GC toxin family.

In addition, GC1 & 2 and GC4 & 5 were evaluated as mixed preparations, although the underlying basis for each combination differs. GC1 and GC2 are known to form a C11 epimeric pair, and the initially purified GC2 partially converted to GC1 prior to the N2a assay. GC1 & 2 have previously been evaluated together as an epimeric mixture [5]. In contrast, GC4 & 5 was found to consist predominantly of GC4, with no clear evidence for GC5. However, because GC4 and GC5 could not be unequivocally differentiated using the present analytical methods, the preparation was conservatively designated GC4 & 5. Consequently, the TEF reported for GC1 & 2 represents the combined functional potency of the two epimers and cannot be assigned to GC1 or GC2 individually, whereas the TEF reported for GC4 & 5 is expected to predominantly reflect the functional potency of GC4. This distinction should be considered when interpreting the apparent structure–activity relationships among the GC toxins evaluated in this study.

Distinct from the acid-mediated hydrolysis of the toxin extract discussed above, the toxicological relevance of GC toxins should also be considered in the context of their fate after transfer from *G. catenatum* to seafood vectors, which proceeds through an enzymatic rather than chemical mechanism. In a related study, Vale (2008) reported that although GC1–GC3 constituted substantial components of the toxin profile in cultured *G. catenatum*, these benzoate analogues were present only at relatively low levels in naturally contaminated bivalves, in which the corresponding decarbamoyl analogues, including dcGTX2+3 and dcSTX, were more prominent [42]. In vitro incubations with bivalve digestive glands further demonstrated conversion of benzoate PSTs into decarbamoyl derivatives via carbamoylase activity, supporting an important role for bivalve biotransformation in modifying the toxin profile encountered by consumers. Thus, the PST composition of the producing dinoflagellate should not be assumed to correspond directly to the toxin composition of contaminated shellfish. This transformation also has practical implications for food-safety monitoring, because several decarbamoyl PSTs are already included in established analytical frameworks and CRMs for conventional and decarbamoyl PSTs are available. Indeed, the study by Vale (2008) [42] used CRMs for STX, dcSTX, dcGTX2+3, and other classical PSTs for identification and quantification. In contrast, CRMs for GC toxins themselves remain unavailable. Therefore, although bivalve biotransformation may reduce the direct contribution of intact GC toxins to human dietary exposure, characterization of their intrinsic functional potency remains relevant for understanding the broader PST profile produced by *G. catenatum* and the toxicological consequences of subsequent transformation.

The present TEFs should therefore be regarded as preliminary and assay-specific estimates rather than definitive toxic equivalence values for regulatory application. Material availability limited each GC toxin preparation to a single N2a assay with triplicate technical wells, and independent biological replication across separate culture, purification, or assay batches was not possible; consequently, inter-assay and between-preparation reproducibility could not be evaluated. The 95% CI reported for the EC_50_ estimates reflect uncertainty associated with nonlinear curve fitting within an individual assay and do not represent variability among independent biological experiments. As noted above, the use of GC1 & 2 and GC4 & 5 precludes attribution of the corresponding TEFs to individual analogues [5], and the tested preparations may not capture the complete GC toxin repertoire of the strain. In addition, the potential biotransformation of GC toxins in bivalves means that these TEFs should not be directly extrapolated to human dietary exposure without considering the toxin composition present in contaminated seafood.

Despite these limitations, the present study demonstrates that purified GC toxins produce distinguishable, concentration-dependent responses in the N2a assay, providing the first functional potency estimates for this class of PST analogues and a practical framework for their toxicological characterization in the continued absence of CRMs. Future studies using independently isolated and quantitatively characterized individual GC toxins, together with electrophysiological, receptor-binding, analytical, and computational approaches, will be required to validate the present TEFs, assess their reproducibility, and establish more definitive structure–activity relationships for GC toxins and other structurally novel PST analogues.

## 5. Conclusions

The present study demonstrated that the N2a assay can be applied to the functional evaluation of PSTs produced by G. catenatum. Using this assay with the G. catenatum extract, the total STX-equivalent functional toxicity was estimated at 3.7275 pg STX eq cell^−1^, confirming that the tested Korean strain produces VGSC-blocking toxins at measurable levels. Structural characterization by NMR and LC-MRM-MS/MS confirmed the identity of the isolated GC1 & 2, GC3, and GC4 & 5 preparations, which showed distinct, concentration-dependent responses in the N2a assay and yielded TEFs in the order GC3 > GC4 & 5 > GC1 & 2. These functional potency estimates differed markedly from previously reported receptor-binding and docking-based predictions, indicating that binding affinity alone does not fully capture cellular functional potency and highlighting the complementary value of cell-based functional assays. Collectively, these findings support the utility of the N2a assay as a complementary tool for evaluating PST-containing algal extracts and GC toxins in the absence of CRMs. Because the present estimates were derived from single N2a assays per preparation under the current experimental conditions, they should be interpreted as preliminary and assay-specific rather than definitive toxic equivalence values. Nevertheless, this study provides the first functional potency estimates for GC toxins and an important starting point for the broader application of functional bioassays to characterize GC toxins and other structurally novel PST analogues, pending validation through independently purified analogues and complementary electrophysiological, receptor-binding, analytical, and computational approaches.

## Figures and Tables

**Table 1:** Quality control criteria for N2a assay.

Parameter	Acceptance Criterion	Description
RCV	≥0.9	Initial N2a cell viability at maximal well density, 24 h after seeding (mean absorbance of *n* = 60 internal wells); confirms high initial cell viability.
COV−	≥0.9	Final cell viability under OV− (untreated) conditions after an additional 24 h incubation; confirms preservation of cell viability in the absence of O/V.
COV+	≤0.3	Final cell viability under OV+ (treated) conditions after an additional 24 h incubation; confirms the expected O/V-induced cytotoxicity.
QCOV−	≥0.9	Final cell viability when cells are treated with STX at the EC_50_ concentration under OV−; confirms the absence of non-specific cytotoxicity from test solution alone.
QCOV+	(0.2 × COV+ + 0.8 × RCV) ≤ QCOV+ ≤ (0.2 × COV+ + 0.8 × RCV)	Final cell viability when cells are treated with STX at the EC_50_ concentration together under OV+; confirms the presence of detectable sodium channel-inhibitory activity.

**Table 2:** Best-fit Hill slope and goodness-of-fit statistics for the OV+ dose–response curves of STX and diluted *G. catenatum* extracts.

Sample	Hill Slope	95% Hill Slope	R^2^
STX	2.615	1.913–3.808	0.984
1/1 extract	ND	–	0.639
1/10 extract	1.409	1.003–1.882	0.985
1/20 extract	1.985	1.760–2.240	0.996

ND: not determined because an acceptable sigmoidal dose–response curve was not obtained.

**Table 3:** EC_50_ values of STX and diluted *G. catenatum* extracts at different dilution ratios, and corresponding STX equivalent toxicity.

Sample	EC_50_	95% CI	STX Equivalent Toxicity (pg STX eq cell^−1^)
STX	2822.5 fg μL^−1^	2479.8–3170.4 fg μL^−1^	Reference Toxin
1/1 extract	ND	ND	ND
1/10 extract	254.2 cells eq mL^−1^	164.7–317.6 cells eq mL^−1^	ND
1/20 extract	757.2 cells eq mL^−1^	709.7–808.2 cells eq mL^−1^	3.7275

ND: not determined because an acceptable sigmoidal dose-response curve was not obtained.

**Table 4:** ^1^H NMR spectral data for GC1–GC4 & 5 in D_2_O with 0.1% acetic acid-*d*_4_ (500 MHz for ^1^H).

Position	GC1 & 2	GC3	GC4 & 5
6	3.99, dd(8.3, 5.1)	3.97, dd(9.1, 5.1)	3.62, dd(11.7, 4.7)
10	3.92, dd(12.0, 4.9)	3.54, m	4.04, d(11.7)
	4.14, d(12.0)	3.77, m	4.66, d(4.7)
11	4.74 *	2.31, m; 2.38, m	4.21, t(5.1)
17	4.27, dd(11.7, 5.1)	4.26, dd(11.7, 5.1)	4.52, dd(12.2, 5.1)
	4.58, dd(11.7, 8.3)	4.60, dd(11.7, 9.1)	4.83 *
2′(6′)	7.94, d(8.6)	7.94, d(8.6)	7.88, d(8.6)
3′(5′)	6.95, d(8.6)	6.95, d(8.6)	6.95, d(8.6)

Data are presented as δ (ppm), multiplicity (*J*_HH_ Hz). *: Overlapped signals.

**Table 5:** EC_50_ values of STX and purified GC toxins determined by the N2a assay, and corresponding TEFs.

Compound	EC_50_ (Assay 1)	95% CI	EC_50_ (Assay 2)	95% CI	TEF
STX (fg μL^−1^)	2822.5	2479.8–3170.4	3278.2	2922.9–3667.8	1.0000
GC1 & 2 (fg μL^−1^)	623,809	587,544–665,583	NA	NA	0.0045
GC3 (fg μL^−1^)	NA	NA	159,151	146,764–171,143	0.0206
GC4 & 5 (fg μL^−1^)	NA	NA	238,384	214,913–262,881	0.0138

The TEF of STX was set to 1.0 in accordance with FAO/WHO (2016) recommendations [27]. NA: not available.

## Data Availability

The data presented in this study are included in the article and Supplementary Materials. Further inquiries can be directed to the corresponding author(s).

## Supplementary Materials

The following supporting information can be downloaded at https://www.mdpi.com/article/10.3390/toxics14090784/s1, Figure S1: Representative HPLC-FLD chromatograms for purified GC1 & 2, GC3, and GC4 & 5; Figure S2. ^1^H NMR spectrum of GC1 or GC2; Figure S3. ^1^H NMR spectrum of GC3(* glycerol); Figure S4. ^1^H NMR spectrum of GC4 or GC5; Figure S5. (A) ^1^H-^1^H COSY and (B) TOCSY spectra of GC3; Figure S6. (A) ^1^H-^1^H COSY and (B) TOCSY spectra of GC4; Figure S7. LC-MRM-MS of GC1–GC4; Table S1: Literature-based comparison of reported toxicity values for *G. catenatum* strains converted to STX equivalents per cell. References [37,38,43,44,45,46,47,48,49,50] are cited in Supplementary Materials.

## Abbreviations

The following abbreviations are used in this manuscript:

HABs	Harmful algal blooms
PST	Paralytic shellfish toxin
PSP	Paralytic shellfish poisoning
VGSCs	Voltage-gated sodium channels
CRMs	Certified reference materials
MBA	Mouse bioassay
HPLC-FLD	High-performance liquid chromatography with fluorescence detection
LC-MS/MS	Liquid chromatography-tandem mass spectrometry
LOD	Limit of detection
STX	Saxitoxin
eq	Equivalent
N2a	Neuro-2a
TEFs	Toxicity equivalence factors
OV	Ouabain octahydrate/Veratridine
EC_50_	Half-maximal effective concentration
FBS	Fetal bovine serum
DPBS	Dulbecco’s phosphate-buffered saline
O	Ouabain octahydrate
V	Veratridine
MTT	thiazolyl blue tetrazolium bromide
ESI	Electrospray ionization
MRM	Multiple reaction monitoring
QC	Quality control
RCV	Reference cell viability
COV−	Viability controls under OV−
COV+	Viability controls under OV+
QCOV−	Quality control samples under OV−
QCOV+	Quality control samples under OV+
