# Supplementary material for: Saxitoxin-Equivalent Toxicity of Gymnodinium catenatum and Toxicity Equivalence Factors of GC Toxins Determined by the Neuro-2a Assay
Source: Toxics. 2026 Sep 4;14(9):784. doi: 10.3390/toxics14090784 (PMC13611359; doi:10.3390/toxics14090784)
Supplement: Supplementary file 1 [file toxics-14-00784-s001.zip › toxics-4486791-supplementary.pdf]

Saxitoxin-Equivalent Toxicity of *Gymnodinium catenatum* and Toxicity Equivalence Factors of GC Toxins Determined by the Neuro-2a Assay

Jeong-In Park [1,2,†], Jun Kim [1,3,†], Youngjin Kim [1,4], Young-Seok Han [5], Jung-Rae Rho [6], Yeonjung Lee [7], Dong Han Choi [7,8,*] and Youn-Jung Kim [1,2,*]

1 Department of Marine Science, Incheon National University, Incheon 22012, Republic of Korea; qkrwjdls0812@naver.com (J.-I.P.); gottkf0167@naver.com (J.K.); yjkim@safety-as.com (Y.K.)
2 Research Institute of Basic Science, Incheon National University, Incheon 22012, Republic of Korea
3 Department of Life Science, Hanyang University, Seoul 04763, Republic of Korea
4 Safety Core Lab Inc., Seoul 04309, Republic of Korea
5 NEB Co., Seoul 08054, Republic of Korea; neb.yshan@gmail.com
6 Department of Oceanography, Kunsan National University, Gunsan 54150, Republic of Korea;
jrrho@kunsan.ac.kr
7 Ocean Climate Response & Ecosystem Research Department, Korea Institute of Ocean Science and
Technology, Busan 49111, Republic of Korea; leey@kiost.ac.kr
8 Department of Convergence Study on the Ocean Science and Technology, Ocean Science and Technology School, Korea Maritime and Ocean University, Busan 49112, Republic of Korea
* Correspondence: dhchoi@kiost.ac.kr (D.H.C.); duckyj@inu.ac.kr (Y.-J.K.)
† These authors contributed equally to this work.

Figure S1. Representative HPLC-FLD chromatograms for purified GC1 & 2, GC3, and GC4 & 5.

Figure S2. ^{1}H NMR spectrum of GC1 or GC2.

Figure S3. ^{1}H NMR spectrum of GC3(* glycerol).

Figure S4. ^{1}H NMR spectrum of GC4 or GC5.

Figure S5. (A)^{1}H-^{1}H COSY and (B) TOCSY spectra of GC3.

Figure S6. (A)^{1}H-^{1}H COSY and (B) TOCSY spectra of GC4.

Figure S7. LC-MRM-MS of GC1–GC4

Table S1. Literature-based comparison of reported toxicity values for *G. catenatum* strains converted to STX equivalents per cell

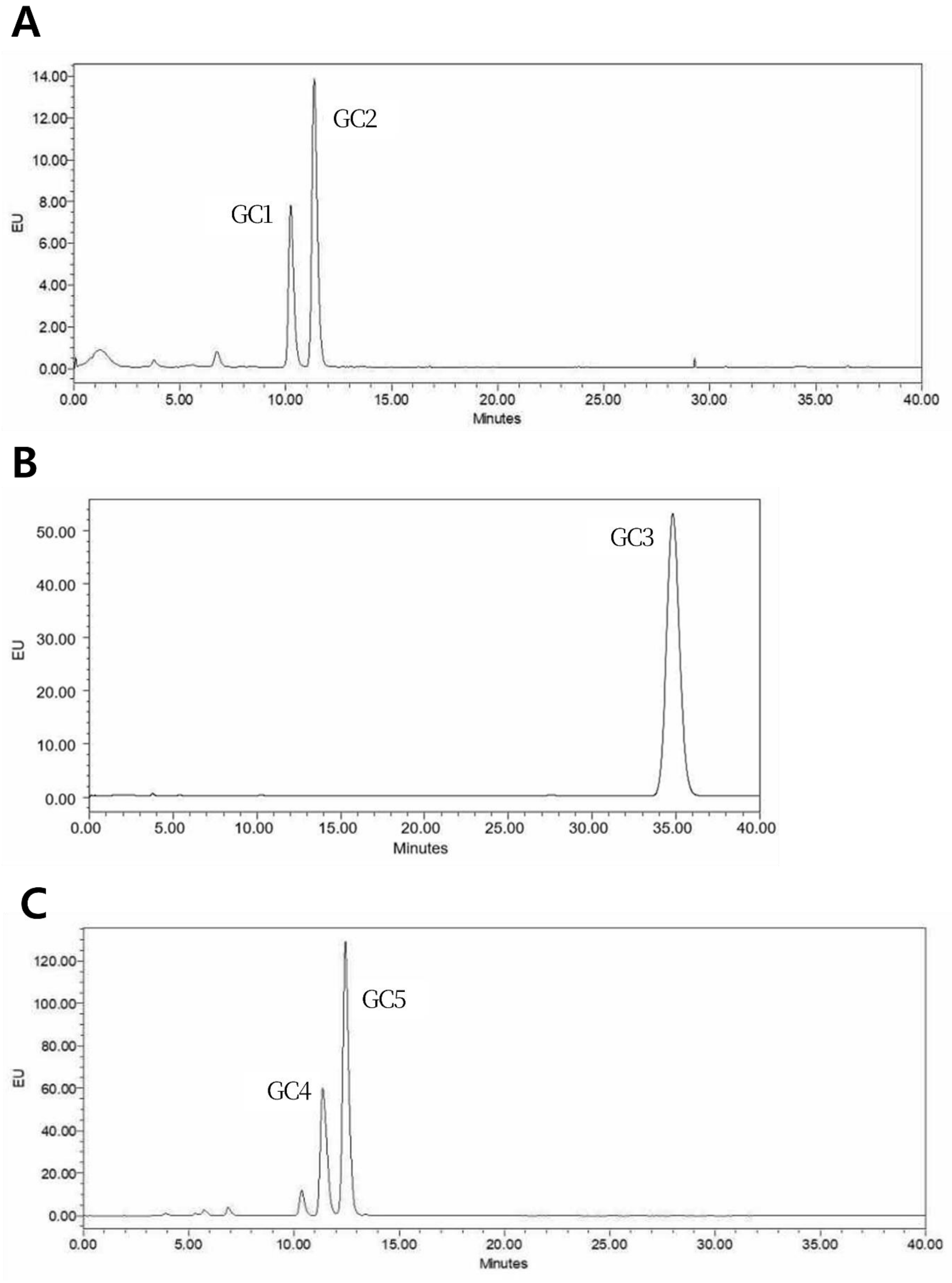

Figure S1. Representative HPLC-FLD chromatograms for purified GC1 & 2, GC3, and GC4 & 5. Peaks corresponding to GC1 and GC2, GC3, and GC4 and GC5 are indicated in each chromatogram.

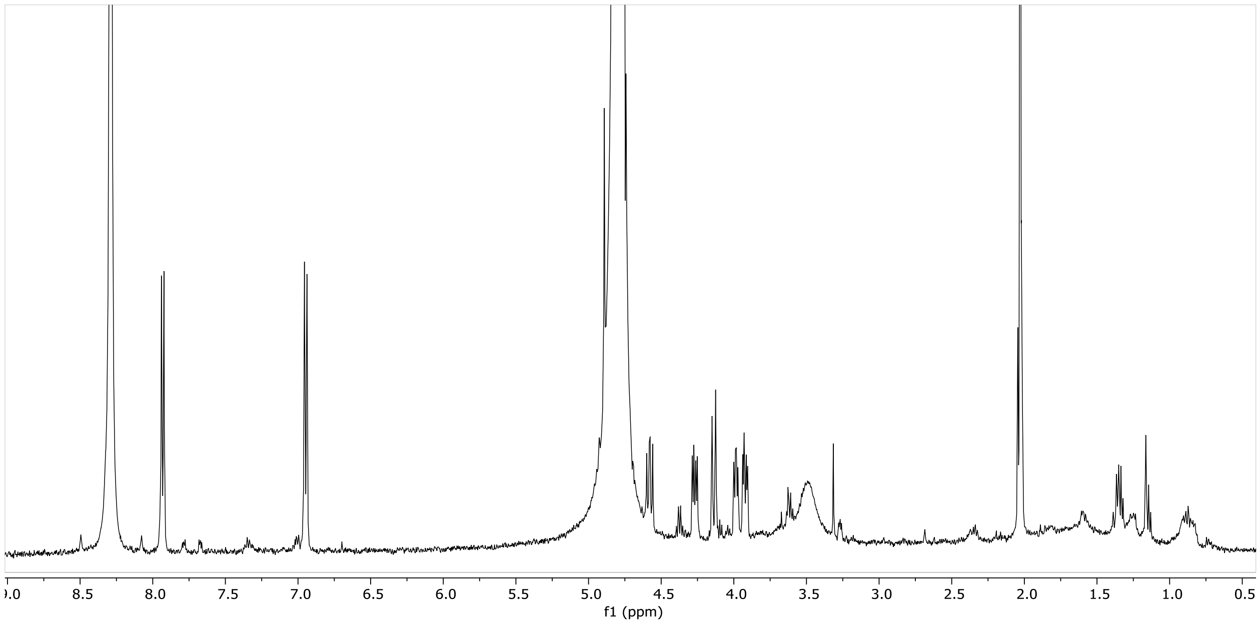

Figure S2. ^{1}H NMR spectrum of GC1 or GC2.

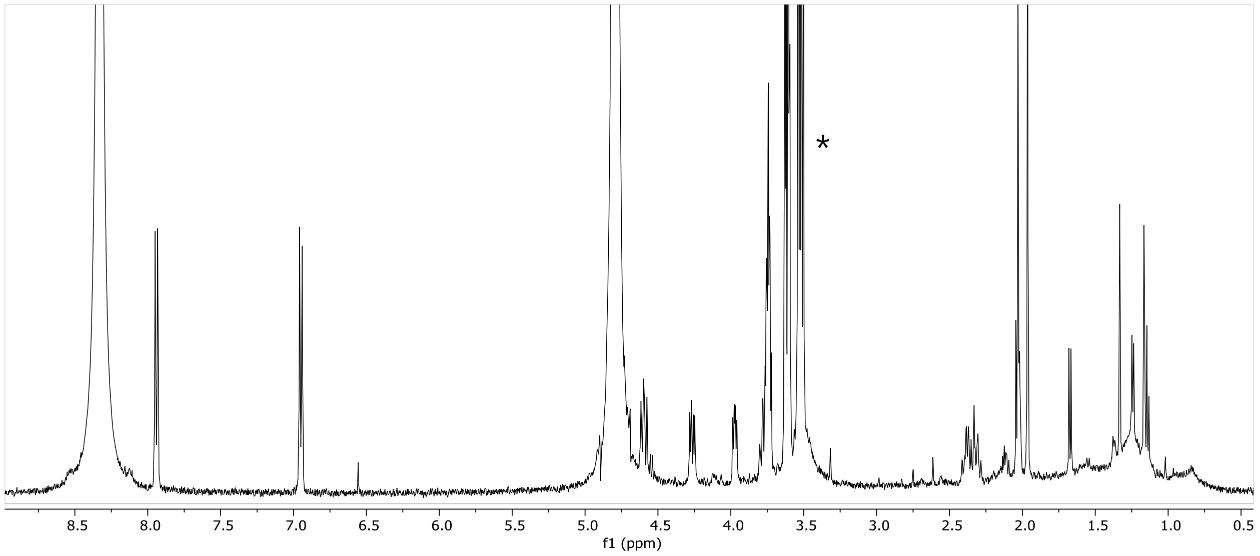

Figure S3. ^{1}H NMR spectrum of GC3 (* glycerol).

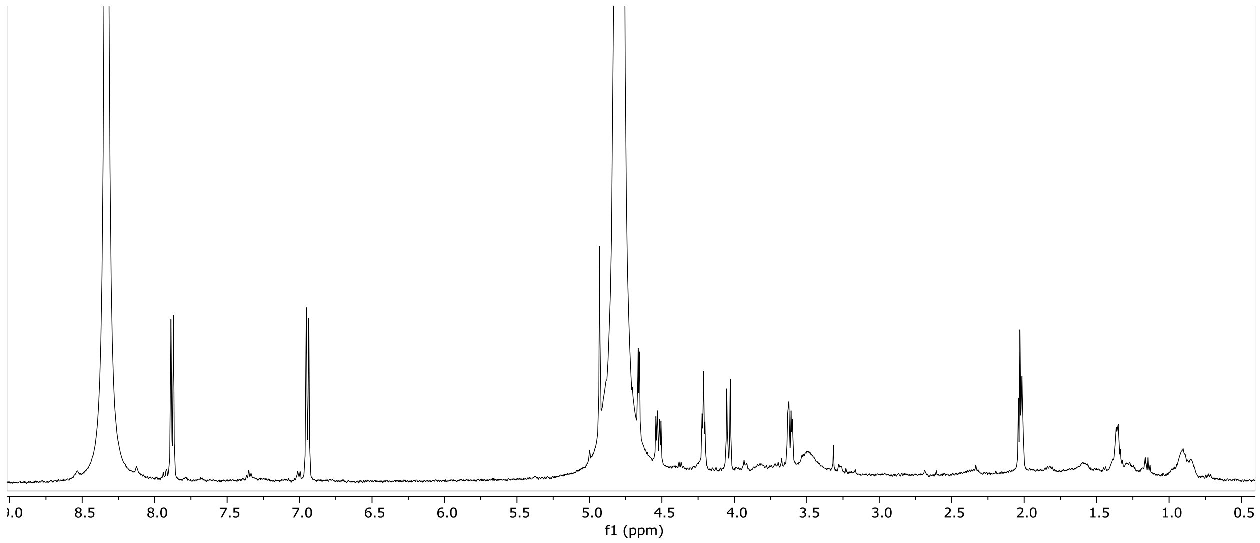

Figure S4. ^{1}H NMR spectrum of GC4 or GC5.

(A)

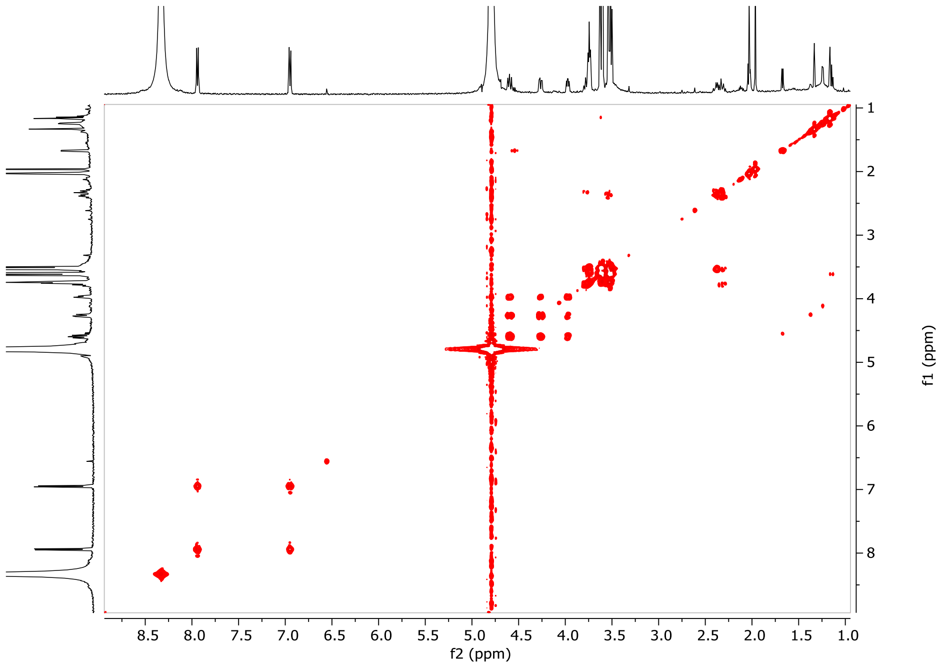

(B)

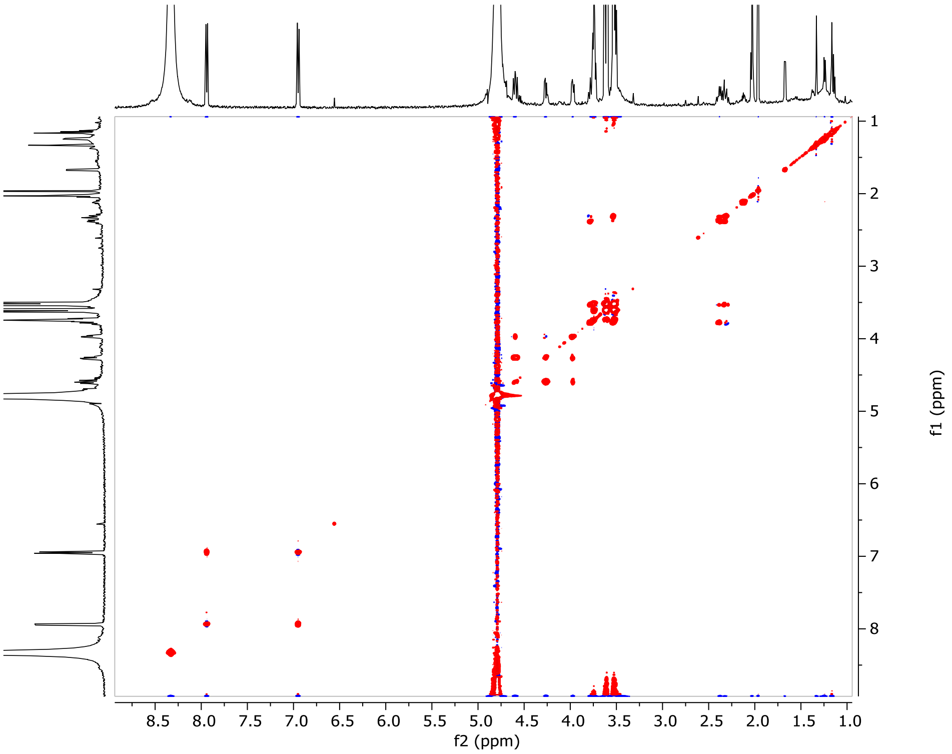

Figure S5. (A)^{1}H-^{1}H COSY and (B) TOCSY spectra of GC3.

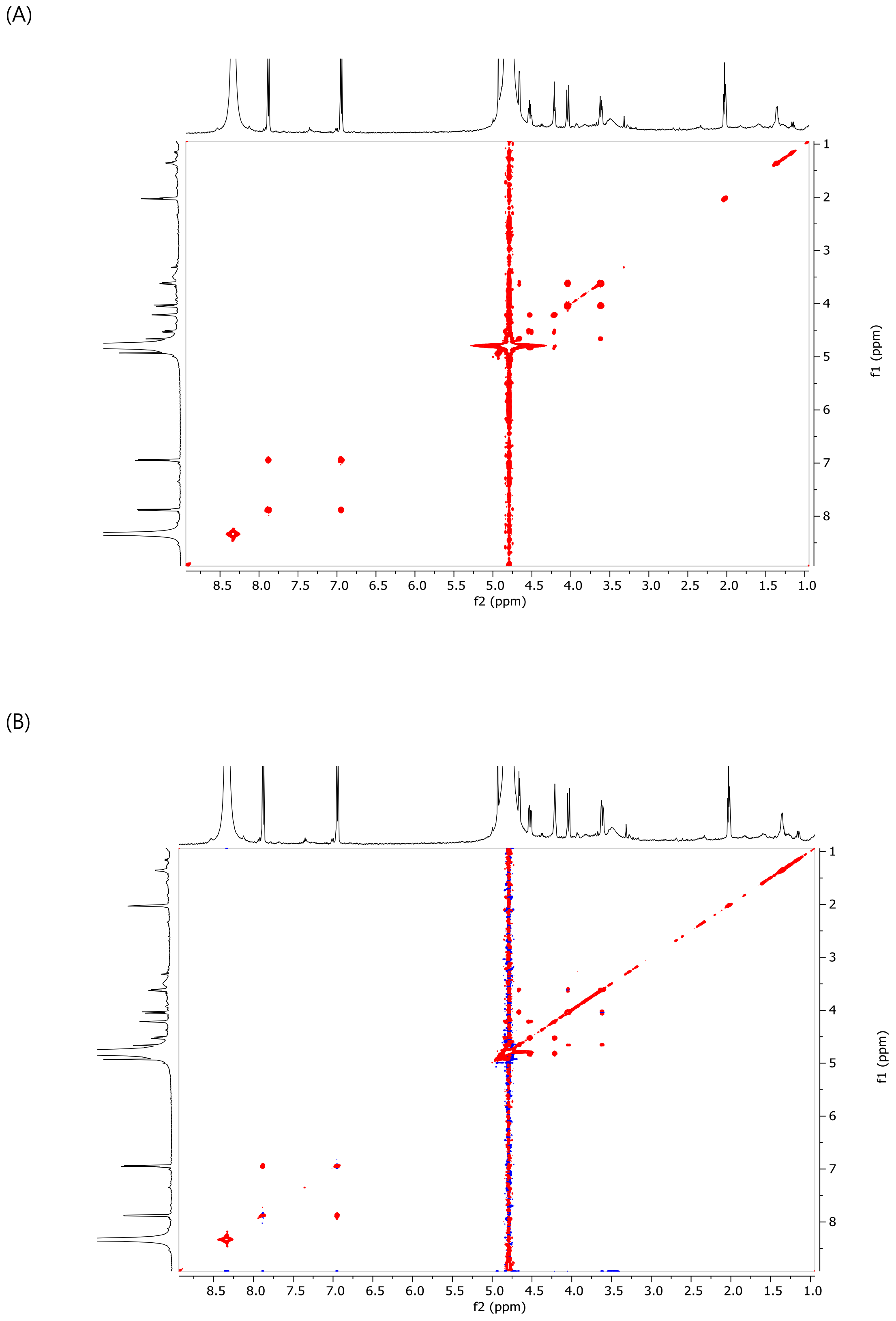

Figure S6. (A)^{1}H-^{1}H COSY and (B) TOCSY spectra of GC4 or GC5.

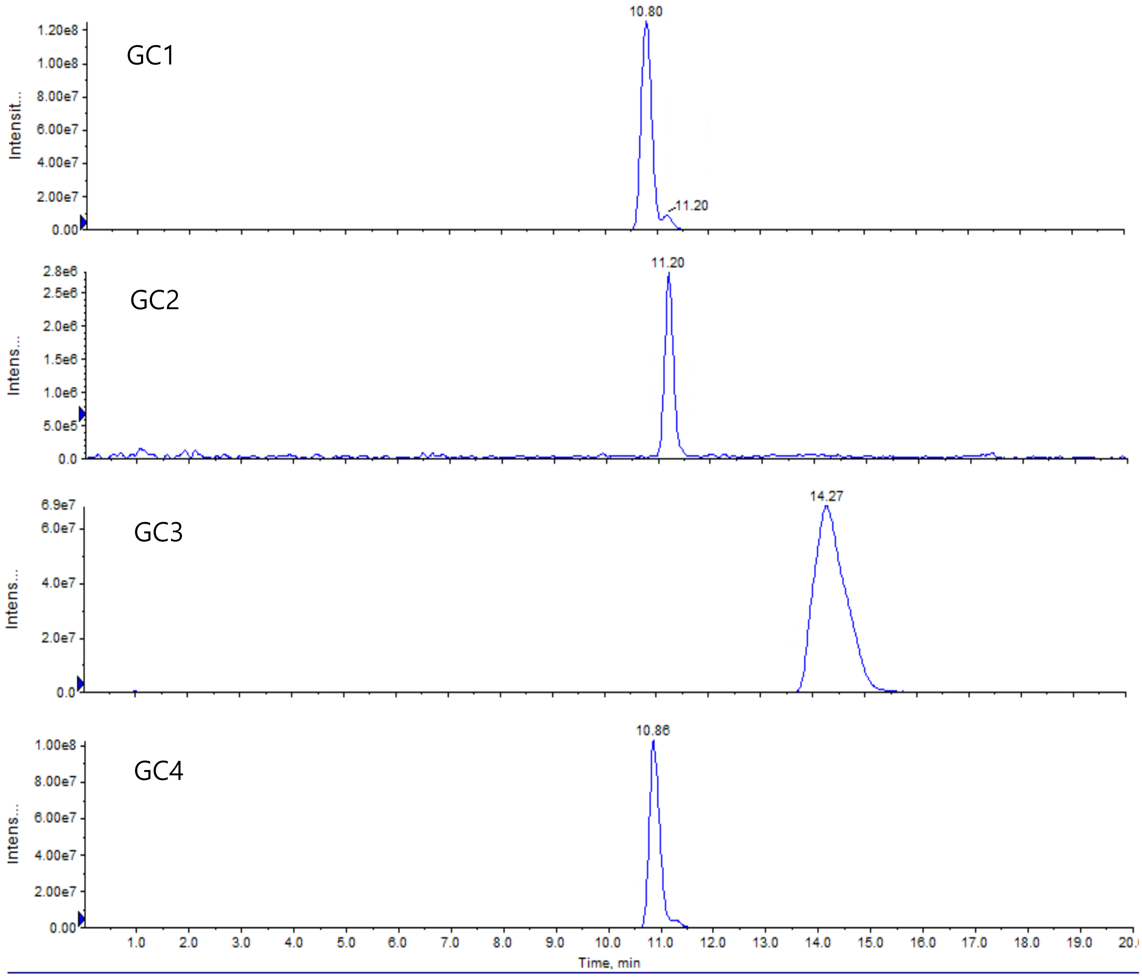

Figure S7. LC-MRM-MS of GC1–GC4

Table S1. Literature-based comparison of reported toxicity values for *G. catenatum* strains converted to STX equivalents per cell.

Location	Detection method	Harvested cell density (cells)	Toxicity (pg STX eq cell-1)	Reference
Tongyeong, Korea	CBA	36,855,000	3.72	In this study
Lázaro Cárdenas, Mexico	HPLC-FLD	333,000	30.00 ± 7.14	[43]
Xiamen Bay, China	HPLC-MS/MS	~644,900 ± 39,500	8.72 ± 0.47 to 12.26 ± 0.10	[38]
Gulf of California, Mexico	HPLC-FLD	22,020 ± 8,250 to 95,640 ± 33,150	4.89-149.81	[44]
Kamae Bay, Japan	HPLC-FLD	~98,780,000 ± 26,170,000	~93.62 ± 10.91	[45]
Pacific coast, Mexico	HPLC-FLD	470,000-550,000	10.10-28.19	[37]
Pacific coast, Mexico	HPLC-FLD	~1,460,000	1.4-10.9	[46]
Bahía de Mazatlán, Mexico	HPLC-FLD	~508,950	13-101	[47]
Yellow Sea, Korea	HPLC-FLD	100,000	17	[48]
South Sea, Korea	HPLC-FLD	100,000	9	
Singapore	HPLC-FLD	100,000	2.99 / 6.67	[49]
Santa Catarina, Brazil	MBA	250,000	28	[50]

Values were compiled from published studies and are presented as reported in the original references (Mean ± SD, range, or single values, depending on the source). Where possible, toxicity values were standardized to STX equivalents per cell based on the original reporting units and analytical procedures. Because analytical methods, culture conditions, growth phases, and extraction protocols differed among studies, these data are provided for contextual comparison only and should not be interpreted as directly comparable. The tilde symbol (~) indicates approximate values reported in the original studies.
